# Enrichment and Evaluation of Antitumor Properties of Total Flavonoids from *Juglans mandshurica* Maxim

**DOI:** 10.3390/molecules29091976

**Published:** 2024-04-25

**Authors:** Shuli Yang, Guodong Chu, Jiacheng Wu, Guofeng Zhang, Linna Du, Ruixin Lin

**Affiliations:** 1 Department of Obstetrics and Gynecology, The Second Hospital of Jilin University, Jilin University, Changchun 130041, China; 2 Engineering Research Center of Bioreactor and Pharmaceutical Development, Ministry of Education, College of Life Science, Jilin Agricultural University, No. 2888, Xincheng Street, Changchun 130118, China; 3 Department of Hepato–Biliary–Pancreatic Surgery, The Second Hospital of Jilin University, Jilin University, Changchun 130041, China

**Keywords:** *Juglans mandshurica* Maxim, resin, flavonoids, enrichment, apoptosis

## Abstract

Flavonoids are important secondary metabolites found in *Juglans mandshurica* Maxim., which is a precious reservoir of bioactive substances in China. To explore the antitumor actions of flavonoids (JMFs) from the waste branches of *J. mandshurica*, the following optimized purification parameters of JMFs by macroporous resins were first obtained. The loading concentration, flow rate, and loading volume of raw flavonoid extracts were 1.4 mg/mL, 2.4 BV/h, and 5 BV, respectively, and for desorption, 60% ethanol (4 BV) was selected to elute JMFs-loaded AB-8 resin at a flow rate of 2.4 BV/h. This adsorption behavior can be explained by the pseudo-second-order kinetic model and Langmuir isotherm model. Subsequently, JMFs were identified using Fourier transform infrared combined with high-performance liquid chromatography and tandem mass spectrometry, and a total of 156 flavonoids were identified. Furthermore, the inhibitory potential of JMFs on the proliferation, migration, and invasion of HepG2 cells was demonstrated. The results also show that exposure to JMFs induced apoptotic cell death, which might be associated with extrinsic and intrinsic pathways. Additionally, flow cytometry detection found that JMFs exposure triggered S phase arrest and the generation of reactive oxygen species in HepG2 cells. These findings suggest that the JMFs purified in this study represent great potential for the treatment of liver cancer.

## 1. Introduction

Nowadays, cancer is a difficult clinical health problem with steadily increasing incidence and mortality [1,2,3,4,5]. This is particularly evident in China, where cancer has become the leading cause of death [6]. It was reported that in 2018 alone, the number of new cancer patients and deaths in China reached almost 4.29 million and 2.87 million, respectively [7]. Liver cancer remains one of the most common types of cancer, and the incidence of liver cancer was estimated to exceed 1 million cases by 2025 [8]. However, although the early diagnosis and treatment of liver cancer are gradually improving, the survival rate of liver cancer patients is still extremely low [9]. Meanwhile, some treatments for liver cancer, such as chemotherapy, have been proven to have serious side effects [10,11]. Thus, there is great interest in finding effective and safe anticancer substances from natural products, especially nutraceuticals and functional foods [12].

*Juglans mandshurica* Maxim. is a perennial tree of *Juglandaceae* widely distributed throughout India, China, and other Asian countries [13]. A large amount of evidence shows that various components with nutritional value are found in this plant, including lipids, proteins, carbohydrates, vitamins, and minerals [14]. In addition to its nutritional function, *J. mandshurica* was also proven to have important medical potential [15]. However, the pruning procedure carried out every year has produced a large number of waste branches of *J. mandshurica*, which are usually burned and discarded, resulting in a great waste of resources and increased environmental pressure. Modern studies have revealed that *J. mandshurica* has the ability to modulate a broad spectrum of pathophysiological processes, such as oxidative stress, inflammation, etc., which are closely related to their various bioactive metabolites [16]. According to existing reports, a variety of compounds have been identified from *J. mandshurica*, which has led to *J. mandshurica* being considered a prominent source of bioactive compounds with potential health benefits [17]. Among many active substances, flavonoids have attracted extensive attention and are considered indispensable substances in the fields of nutrition and pharmacy due to their wide biological activities and therapeutic potential [18,19].

Flavonoids are polyphenolic metabolites with low molecular weight and are widely found in plants and fungi [20]. Pharmacological studies have confirmed the anticancer activity of flavonoids derived from a variety of plants, including *Celastrus hindsii*, *Licorice*, *Apium graveolens*, etc. [21,22]. In addition, according to reports, the side effects of flavonoids obtained from plants are very low [23]. Hence, flavonoids are also considered to be used as chemopreventive or chemotherapeutic agents [24,25]. Although *J. mandshurica* has been widely studied, most of the studies have focused on anthraquinones, naphthoquinones, and other components, and few studies have been undertaken about the antitumor activities and mechanisms of its total flavonoids [26]. Thus, with the many medicinal benefits of flavonoids, it seems that the purification of flavonoids from *J. mandshurica* and investigation into their anticancer activities are necessary.

A variety of enrichment methods have been used to purify flavonoids from natural plants, including high-speed counter-current chromatography, silica-gel chromatography, membrane separation, macroporous resin purification, etc. [27]. Among them, macroporous resin purification is considered to be an effective, stable, and environment-friendly method to adsorb and purify flavonoids, which relies on its high capacity of adsorption and desorption, reusability, large surface area, and long service period. A number of flavonoids from a variety of products, including *Brassica oleracea* and *Pteris ensiformis*, have been enriched and purified by macroporous resins [28,29]. Hence, the establishment of suitable purification conditions for resins is conducive to the further study of flavonoids derived from *J. mandshurica*.

To investigate the potential of total flavonoids (JMFs) from *J. mandshurica* in the treatment of cancer, the present study focused on the establishment of purification conditions suitable for the preparation of JMFs by macroporous resins. Furthermore, JMFs were qualitatively analyzed using Fourier transform infrared (FITR) and high-performance liquid chromatography and tandem mass spectrometry (HPLC-MS/MS). Finally, the anticancer activity and possible mechanisms of JMFs were further studied through flow cytometry, Western blot, etc. Our findings provide experimental evidence for the clinical use of total flavonoids found in *J. mandshurica* as an effective anticancer agent.

## 2. Results and Discussion

### 2.1. Screening the Suitable Macroporous Resin for JMFs Purification

**Figure 1:**
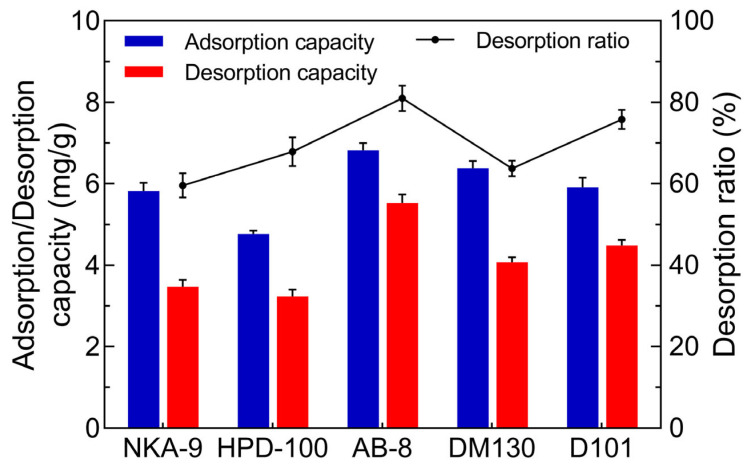
Adsorption and desorption abilities of different resins towards flavonoids extracted from *J. mandshurica*.

Macroporous resins have been considered useful for the purification of various active natural products, including polyphenols, glycosides, and so on. However, the adsorption capacity of macroporous resins is closely related to their surface properties, pore size, adsorbed substrate, etc. [20]. Hence, for realizing the effective enrichment of JMFs, the adsorption capacity and the desorption capacity of tested resins toward JMFs were analyzed. It can be seen in Figure 1 that there was a difference in the adsorption and desorption capacities for the JMFs of resins with different physical and chemical properties, in which polar or weak polar resins had a relatively higher adsorption capacity for JMFs than non-polar resins. Similar results were also found in the research reported by Hou et al. [29]. Compared to NKA-9 and DM130, AB-8 resin possessed a relatively higher adsorption capacity (6.82 mg/g) toward JMFs. The low adsorption capacity of NKA-9 and DM130 may be explained by their small pore size or surface area. Meanwhile, the highest desorption capacity was found in AB-8 (5.52 mg/g), the desorption ratio of which was 80.99%. Considering the significant adsorption and desorption ability, among the resins investigated here, the AB-8 resin was considered to be the resin most conducive to the purification of JMFs.

### 2.2. Adsorption Kinetics of the AB-8 Resin

**Figure 2:**
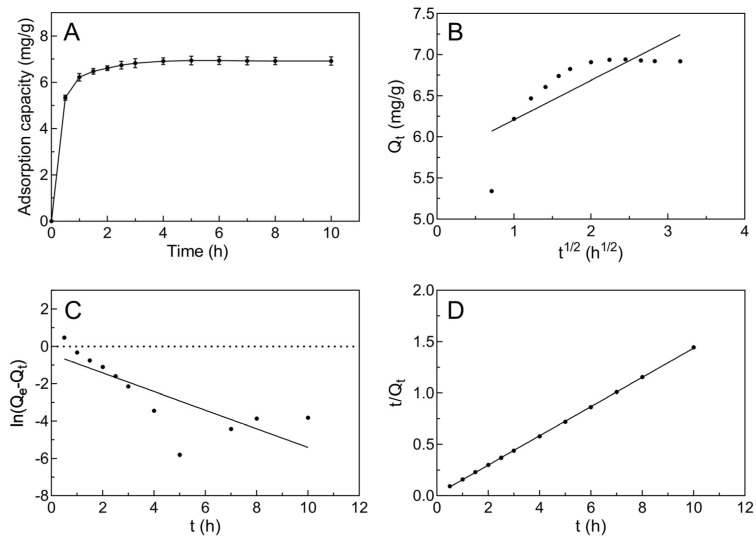
The adsorption kinetics of JMFs on AB-8 resin at 25 °C. (**A**) Adsorption kinetic curve for JMFs sorption onto AB-8 resin. (**B**) Intra-particle diffusion model of JMFs on AB-8 resin. (**C**) Pseudo-first-order model of JMFs on AB-8 resin. (**D**) Pseudo-second-order model of JMFs on AB-8 resin.

For understanding the adsorption process of AB-8 resin for JMFs in the adsorption time from the beginning to equilibrium, the adsorption kinetics of JMFs on AB-8 resin was determined. As exhibited in Figure 2A, the adsorption capacity of the AB-8 resin for JMFs increased quickly in the first hour, and then the increasing trend slowed down with a further increase in the adsorption treatment time. Meanwhile, the adsorption capacity of the selected resin likely reached equilibrium in 5 h, indicating that the surface adsorption sites of the AB-8 resin were almost saturated with JMFs.

Figure 2B–D shows the fitting results of the three kinetic models, and Table 1 shows the adsorption kinetic parameters of the AB-8 resin. From the fitting results of the intra-particle diffusion model, it can be seen that *C* was positive, that is, the slope was greater than zero. It can be inferred that the adsorption behaviors of AB-8 resin for JMFs were intra-particle diffusion and boundary layer diffusion [30]. By calculating the fitting results of different models, the theoretical *Q*_e_ values of the pseudo-first-order model and the pseudo-second-order model were 0.07 mg/g and 7.04 mg/g, respectively. Compared to the pseudo-first-order model, the difference between the theoretical *Q*_e_ value of the pseudo-second-order model and the actual *Q*_e_ value (6.94 mg/g) was smaller. In addition, the *R*^2^ value of the pseudo-second-order model was 0.9999, which is obviously higher than that of the pseudo-first-order model (0.6239). Therefore, it was most suitable to use the pseudo-second-order model to understand the adsorption mechanism of JMFs by AB-8 resin.

### 2.3. Adsorption Isotherm Curve of Selected Macroporous Resin

In order to better understand the interaction between AB-8 resin and JMFs, three adsorption isotherm equations (Langmuir, Freundlich, and Temkin) were used to simulate the whole adsorption process.

**Figure 3:**
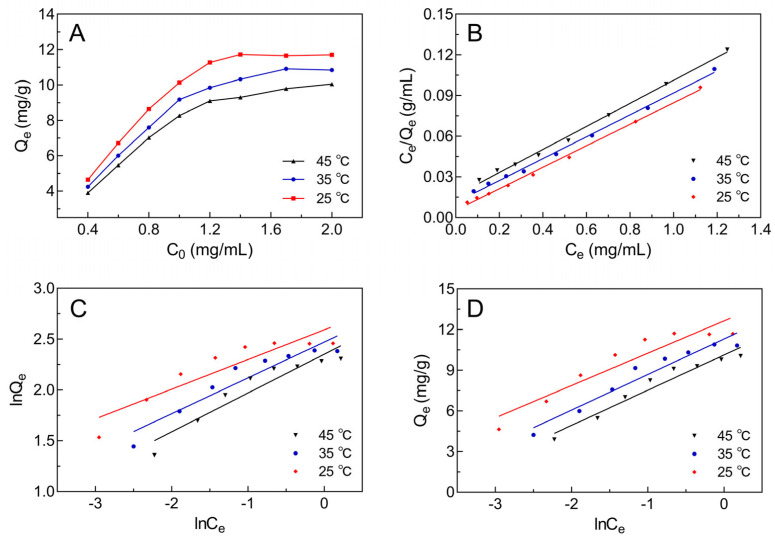
The adsorption isotherms for JMFs on selected resin at different temperatures. (**A**) Adsorption isotherms for JMFs on selected resin at 25 °C, 35 °C, and 45 °C. (**B**) Langmuir adsorption isotherms for JMFs on selected resin. (**C**) Freundlich adsorption isotherms for JMFs on AB-8 resin. (**D**) Temkin isotherm adsorption isotherms for JMFs on AB-8 resin.

Figure 3A shows that the adsorption capacity of the AB-8 resin was enhanced by increasing the initial concentration of JMFs. Additionally, there was a negative correlation between the adsorption temperature and the adsorption capacity of the resin toward JMFs. Thus, the adsorption of the JMFs by the AB-8 resin was more favorable at 25 °C. At 25 °C, the maximum adsorption capacity of JMFs was found at the initial flavonoid concentration of 1.4 mg/mL, which was selected for use in further studies.

The fitting results of the three isotherm models and the adsorption isotherm parameters of the AB-8 resin to flavonoids at different temperatures are portrayed in Figure 3B–D and Table 2, respectively. In the Langmuir isotherm model, the adsorption sites on the surface of the resin are a uniform monolayer, and the adjacent molecules do not interact with each other. In addition, a stronger adsorption performance of the resin appeared at a higher *Q*_m_ value [31]. In the present study, the maximum *Q*_m_ value of the AB-8 resin for JMFs was 12.61 mg/g at 25 °C. It can be seen in Table 2 that the *Q*_m_ value was negatively correlated with the temperature used in adsorption, indicating that the process of the adsorption of JMFs by the AB-8 resin was exothermic. In the Freundlich isotherm model, the resin surface is non-uniform and the adjacent molecules may interfere with each other. In this model, the *K_F_* value was negatively correlated with the adsorption temperature, suggesting that exothermic adsorption was established. Meanwhile, the ratio of 1/n was less than 0.5, indicating that the AB-8 resin had strong adsorption of the JMFs. In the Temkin isotherm model, *K_T_* is the equilibrium constant reflecting the binding ability between the resins and the adsorbed materials. The results show that the *K_T_* value decreased rapidly from 201.64 mL/mg to 49.36 mL/mg with increasing temperatures, indicating that the higher the temperature was, the weaker the binding force between the AB-8 resin and the JMFs was. In other words, the increase in temperature was not conducive to the adsorption of JMFs. Moreover, relative to the Freundlich isotherm and Temkin isotherm, the Langmuir isotherm showed a higher *R*^2^. Hence, it was accepted that the Langmuir isotherm model was a more suitable model to explain the uniform monolayer adsorption mechanism of AB-8 resin to JMFs (Table 2).

### 2.4. Dynamic Adsorption and Desorption Curves on the AB-8 Resin towards JMFs

#### 2.4.1. Screening the Suitable Adsorption Conditions

**Figure 4:**
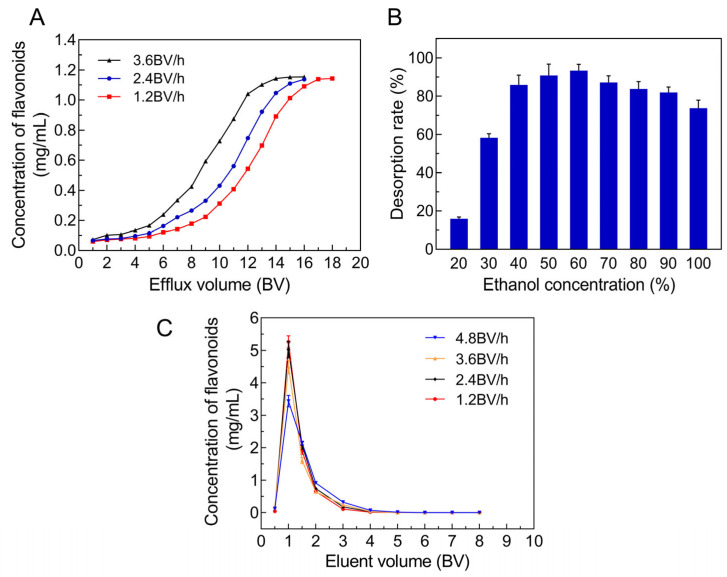
Optimization of adsorption and desorption conditions of JMFs on AB-8 resin. (**A**) Dynamic leakage curves. (**B**) Screening of optimal ethanol concentration for desorption of JMFs on AB-8 resin. (**C**) Dynamic desorption curves.

The dynamic leakage curves of the JMFs on the selected resin were established based on the efflux volume and concentration of JMFs in the effluent. Along this curve, the leakage point was identified once the flavonoid content in the effluent reached one-tenth of the initial loading concentration [32]. As shown in Figure 4A, the leakage curves of three different flow rates show a similar trend. For different flow rates, the flavonoid contents in the effluent increased gradually with continuous elution and finally remained constant. For a flow rate of 1.2 BV/h, 2.4 BV/h, and 3.6 BV/h, the leakage point occurred when the loading volumes were 6 BV, 6 BV, and 4 BV, respectively. Meanwhile, under the same effluent volume, the concentration of flavonoids in the effluent was directly proportional to the flow rate, which may have been caused by the reduction of the contact time between the JMFs and the adsorbent due to a high flow rate. Considering the cost and the effectiveness of the adsorption, the flow rate and the loading volume of the JMFs were set at 2.4 BV/h and 5 BV, respectively.

#### 2.4.2. Screening the Suitable Desorption Conditions

Whether the ethanol concentration affected the desorption of the JMFs was investigated first (Figure 4B). The highest desorption rate was found at a 60% (*v*/*v*) eluted ethanol concentration, which was used in the subsequent purification process.

Then, in order to further select the appropriate desorption flow rate and the volume of eluted ethanol, the dynamic desorption curves were determined at flow rates of 1.2, 2.4, 3.6, and 4.8 BV/h. As presented in Figure 4C, the best elution efficiency was obtained at an elution flow rate of 1.2 BV/h. When the elution flow rates were set to 1.2 and 2.4 BV/h, once the elution volume reached 4 BV, the flavonoids were almost completely eluted from the adsorption column, while under the same elution volume, a small amount of flavonoids had not been eluted at the flow rates of 3.6 and 4.8 BV/h. Therefore, the best flow rate and ethanol volume in the desorption process were set to 2.4 BV/h and 4 BV to improve the efficiency of the desorption.

#### 2.4.3. Validation Experiments

To evaluate the reliability of the proposed purification process, five parallel validation tests were carried out. The purity of the JMFs was 60.85 ± 2.55%, which is 3.46 times higher than that of the crude extracts (17.60 ± 1.41%). The recovery of the total flavonoids was calculated to be 76.4 ± 2.1%.

### 2.5. FTIR Analysis of Enriched JMFs

**Figure 5:**
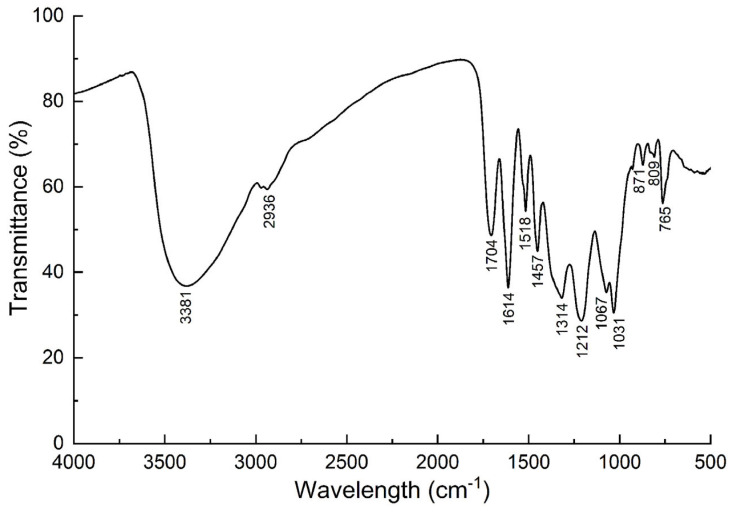
Fourier transform infrared (FTIR) spectra of JMFs.

The FTIR spectrum of JMFs is shown in Figure 5. A broad absorption peak was found at 3381 cm^−1^, which may be related to the stretching vibration of the O-H group in the JMFs. The peak at 2936 cm^−1^ indicates the presence of C-H in the aliphatic and aromatic groups. Characteristic peaks occurred at 1457 cm^−1^ and corresponded to the stretching vibration of C-O. The adsorption peaks of the deformation vibrations of the C-C bands that exist in flavonoids were identified at 1067 cm^−1^ and 1031 cm^−1^ [20,33].

### 2.6. HPLC-MS/MS Analysis of Enriched JMFs

**Figure 6:**
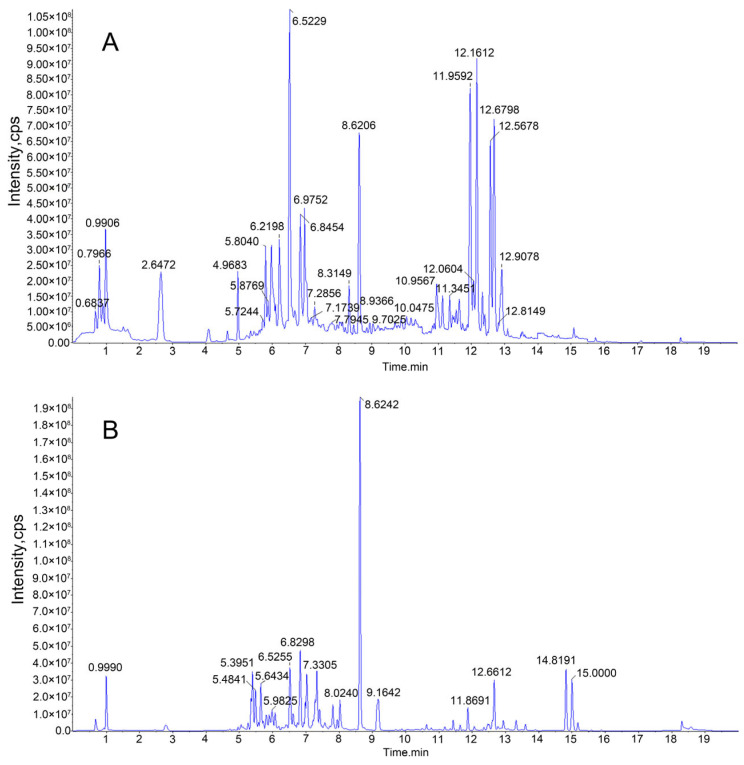
HPLC-MS total ion chromatograms in positive (**A**) and negative (**B**) modes of JMFs.

The purified JMFs were subsequently identified by HPLC-MS/MS. The total ion chromatograms in the positive and negative modes of JMFs are shown in Figure 6. A total of 156 flavonoids were identified from the JMFs, including 6 chalcones and dihydrochalcones, 21 flavanones, 43 flavonols, 59 flavonoids, 13 anthocyanins, and 14 isoflavones. The structure backbone and HPLC-MS detection data of these identified compounds are displayed in Table 3 and Table S1 (Supplementary Materials), respectively. Among the 156 flavonoids identified, most of them exist in the form of free forms or glycosides [23]. In addition, to the best of my knowledge, fewer than 50 flavonoids have been identified from different tissues of *J. mandshurica* [17].

### 2.7. Anticancer Potential of Purified JMFs

#### 2.7.1. Anti-Proliferation Effect of JMFs on HepG2 Cells

**Figure 7:**
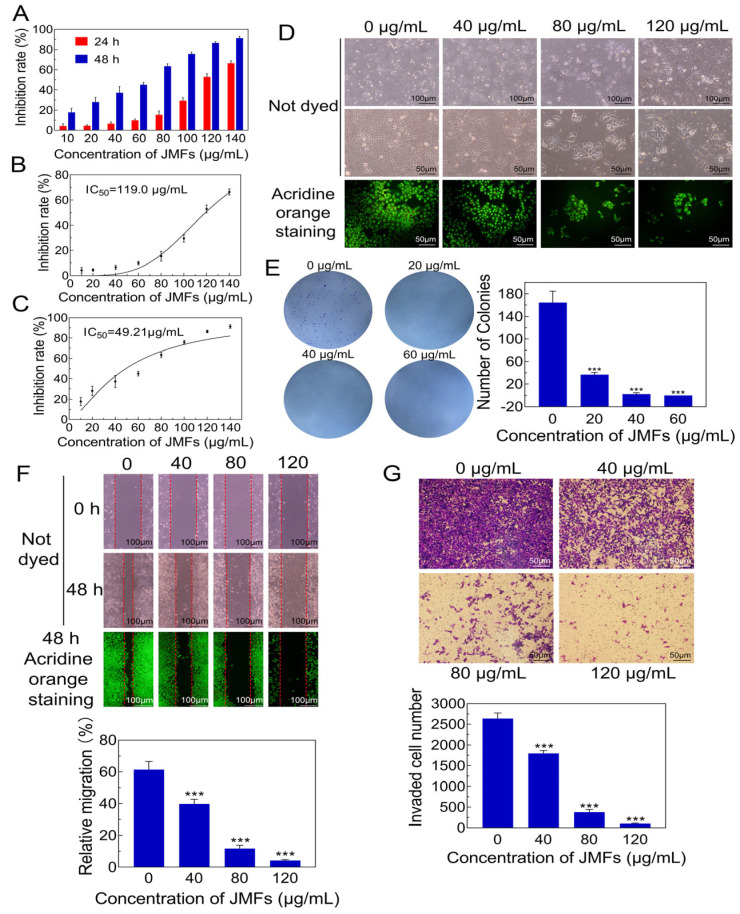
The antitumor efficacy of JMFs. (**A**) The antiproliferative potential of JMFs on HepG2 cells. (**B**) The inhibition curve of JMFs on HepG2 cells was drawn after 24 h treatment. (**C**) The inhibition curve of JMFs on HepG2 cells was drawn after 48 h treatment. (**D**) The morphological changes of HepG2 cells incubated with JMFs for 48 h. (**E**) The effect of JMFs on the colony-forming ability of HepG2 cells and statistical results of the number of corresponding colonies. (**F**) Representative images and quantification results of migration test. (**G**) Effect of JMFs exposure on the invasive migration behavior of HepG2 cells and the quantitative analysis of the number of invasive cells. *** *p* < 0.001 vs. control group (0 μg/mL JMFs).

Flavonoids from some traditional Chinese medicines have been reported to inhibit the proliferation of tumor cells [34]. Hence, the anti-proliferation effect of JMFs on HepG2 cells was also assessed using a cell counting kit 8 (CCK8). It can be seen in Figure 7A that the inhibition potential of JMFs on the viability of HepG2 cells was dramatically enhanced by increasing the JMFs concentrations from 10 to 140 μg/mL. In this assay system, the IC50 values of the JMFs against HepG2 cell proliferation were 119.00 and 49.21 μg/mL for 24 h and 48 h of incubation, respectively (Figure 7B,C). These results imply that the addition of JMFs exhibited anti-proliferation activity against HepG2 cells in a dose-dependent manner. Additionally, the effects of JMFs with different concentrations on the proliferation of normal human hepatocytes (HL-7702 cells) were also detected. Results show that JMFs with concentrations of 10, 20, 40, 60, 80,100, 120, and 140 μg/mL do not affect the proliferation of HL 7102 cells (Figure S1 from Supplementary Materials), indicating that the toxicity of the samples to normal cells within this concentration range is almost negligible.

#### 2.7.2. Morphological Changes in JMFs-Exposed HepG2 Cells

Morphological changes were observed in HepG2 cells after 48 h of JMFs treatment (Figure 7D). It can be seen that the cells without JMFs treatment had regular fusiform, and the cells were closely arranged and grew densely [35]. On the contrary, abnormal cell morphology was clearly observed in the cells treated with different concentrations of JMFs. Meanwhile, a marked decrease in the number of cells was also found. This phenomenon was particularly evident in the cells incubated with higher concentrations of JMFs. Additionally, the cells were also stained with acridine orange to detect the nuclear morphology. The nuclei without JMFs treatment showed intact nuclei, while some cells treated with JMFs showed obvious chromatin condensation or nuclear fragmentation. These results suggest that the JMFs treatment seriously affected the normal morphology of the HepG2 cells.

#### 2.7.3. Inhibitory Potential of JMFs on Colony Formation of HepG2 Cells

A colony-forming assay was employed to assess the long-term effect of JMFs on the survival of HepG2 cells. As illustrated in Figure 7E, fewer and smaller colonies were observed in the JMFs treatment group in relation to the control group (0 μg/mL). The HepG2 cells could hardly form colonies when the concentration of JMFs was higher than 40 μg/mL, which was further proved by the quantification analysis of the number of colonies. Similarly, relative to the control group, the colony-forming ability of JMFs-exposed cells decreased significantly (*p* < 0.001), which further confirms the inhibition property of JMFs against the proliferation of HepG2 cells.

#### 2.7.4. JMFs Inhibited Migration and Invasion Abilities of HepG2 Cells

Various studies have proposed that uncontrolled metastasis is often considered to be the main characteristic of malignant tumors [36]. The ability for migration and invasion is very important for tumor cells to spread to surrounding tissues and even to the whole body, which further affects the therapeutic effects on tumors [37]. Hence, it is necessary to identify whether JMFs could influence the migration and invasion abilities of HepG2 cells. The migration of HepG2 cells was limited by JMFs in a dose-dependent manner (Figure 7F). Relative to the control group, the JMFs-treated cells showed lower migration (*p* < 0.001). Similarly, the results of the invasion assay show that the invaded HepG2 cells markedly decreased after exposure to JMFs compared to the 0 μg/mL JMFs-treated group (*p* < 0.001, Figure 7G). Taken together, these findings imply that the JMFs were capable of influencing the migration and invasion of the HepG2 cells.

#### 2.7.5. JMFs Exposure Triggered S Phase Arrest in HepG2 Cells

**Figure 8:**
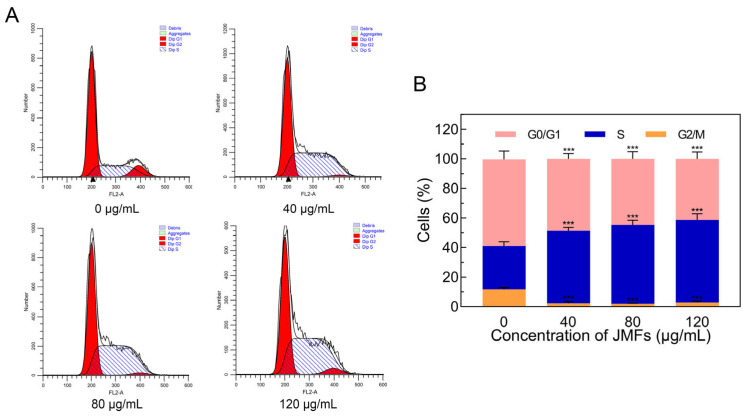
JMFs induced the arrest of cell cycle in HepG2 cells. (**A**) Flow cytometry was employed to detect the cell cycle distribution of JMFs-exposed HepG2 cells. (**B**) The proportion of cell cycle distribution. *** *p* < 0.001 vs. control group (0 μg/mL JMFs).

In the process of cell proliferation, cells need to pass through different distinct phases, including the G1 phase, S phase, G2 phase, and M phase. Disrupting the normal cell cycle is also considered to be a target for cancer treatment [38,39]. To clarify the underlying mechanisms of the inhibitory action of JMFs on HepG2 proliferation, the cell cycle distribution was investigated by flow cytometry with PI staining. As shown in Figure 8, compared to the untreated cells, an evident increase in the number of cells in the S phase was found in the JMFs-treated cells, while this phenomenon was more obvious with the increase in the JMFs treatment concentration (*p* < 0.001). In contrast, the proportion of cells in the G0/G1 and G2/M phases in the JMFs group was markedly less than that in the control group (*p* < 0.001). Thus, the HepG2 cells treated with JMFs were blocked in the S phase, which may have been closely related to the inhibition of cell proliferation by the JMFs.

#### 2.7.6. Changes in ROS Levels in HepG2 Cells Treated with JMFs

**Figure 9:**
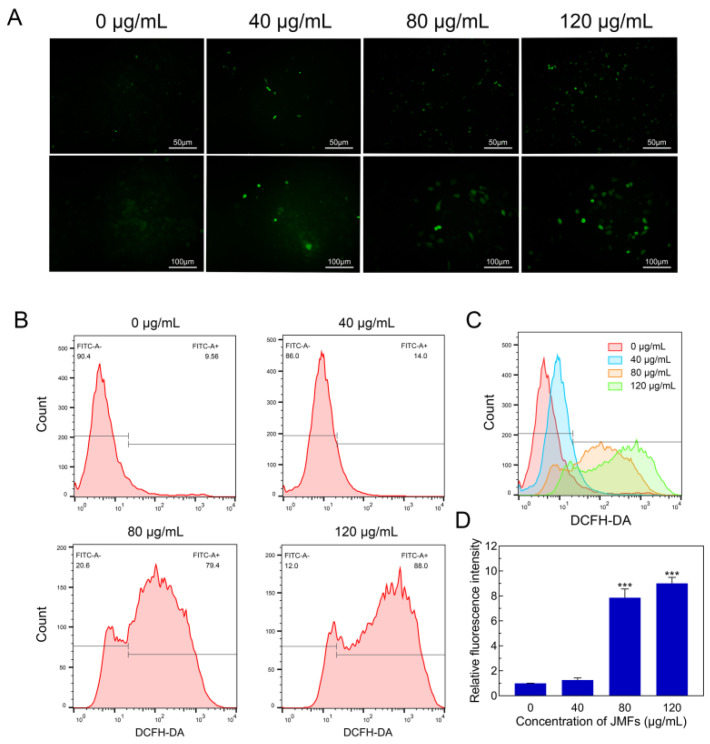
Effect of JMFs incubation on ROS generation in HepG2 cells. (**A**) Images of ROS levels in HepG2 cells after 48 h incubation with JMFs was acquired by fluorescence microscopy coupled with DCFH-DA staining. (**B**) Induction of ROS generation in HepG2 cells incubated with JMFs was determined by flow cytometry. (**C**) The effects of different concentrations of JMFs on the changes in the fluorescence intensity of HepG2 cells. (**D**) Quantification of relative fluorescence intensity of cells treated with JMFs. *** *p* < 0.001 vs. control group (0 μg/mL JMFs).

Recently, increasing the level of reactive oxygen species (ROS) or inhibiting antioxidant capacity, thereby inducing oxidative stress, has been considered an effective anticancer strategy. As a by-product of normal cell metabolism, ROS can cause cell death and tissue damage, especially at high concentrations [40]. Although the antioxidant capacity of tumor cells is higher than that of normal cells, the excessive accumulation of ROS can also disrupt redox homeostasis and cause serious damage to cancer cells [41]. Meanwhile, the generation of intracellular ROS induced by flavonoids was proven to induce tumor cell apoptosis [42]. Microscopy analyses showed that there was a dose-dependent increase in ROS generation in HepG2 cells treated with JMFs (Figure 9A). Furthermore, flow cytometry detection and quantitative analyses also confirmed the role of JMFs treatment in increasing ROS accumulation in HepG2 cells. When the treatment dose of JMFs increased to 80 and 120 μg/mL, the levels of ROS generation in the cells were 8.17 and 9.22 times higher, respectively, than that in the control cells (Figure 9B–D). This suggests that JMFs triggered the accumulation of ROS in tumor cells in a dose-dependent manner, which may have further led to cell damage and apoptosis.

#### 2.7.7. Induction of Apoptosis of HepG2 Cells by JMFs

**Figure 10:**
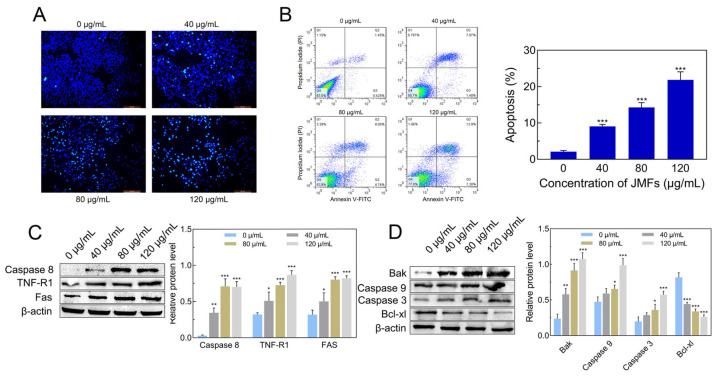
Determination of apoptosis in JMFs-exposed HepG2 cells. (**A**) The apoptosis in HepG2 cells incubation with JMFs was detected by fluorescence microscope after Hoechst 33342 staining. (**B**) Detection of apoptosis rate of HepG2 cells treated with JMFs by flow cytometry. (**C**,**D**) Western blot was employed to determine the expressions of related proteins in JMFs-exposed HepG2 cells in extrinsic and intrinsic pathways. * *p* < 0.05, ** *p* < 0.01, and *** *p* < 0.001 vs. control group (0 μg/mL JMFs).

For exploring whether the JMFs inhibited cell growth via the induction of apoptosis, Hoechst 33342 staining was employed to detect the promoting effect of JMFs on the apoptosis of HepG2 cells. In this test, the nuclei were dyed dark blue in the untreated cells (Figure 10A). However, the number of HepG2 cells dyed dark blue decreased gradually with increases in the JMFs treatment concentration. Moreover, a large number of apoptotic cells represented by bright blue fluorescence were seen in each JMFs treatment group. It can also be seen that the higher concentration of JMFs was, the more apoptotic cells there were. The morphological observation results suggest that the JMFs caused abnormalities in the nucleus and genetic materials of the HepG2 cells, which may have been due to the occurrence of apoptosis induced by the JMFs. Furthermore, the apoptosis of the HepG2 cells was also determined using flow cytometry. After 48 h of culture, the apoptosis rate of the control group was only 2.12%, while the apoptosis rates of the cells treated with JMFs at concentrations of 40, 80, and 120 μg/mL increased to 9.05%, 14.27%, and 21.83%, respectively (Figure 10B). Taken together, JMFs can induce apoptosis in HepG2 cells and inhibit cell growth.

Apoptosis is a complex process involving multiple signal pathways, including extrinsic and intrinsic pathways [43]. For understanding the effect of JMFs on the related proteins in these two pathways, Western blot detection was employed. As demonstrated in Figure 10C, JMFs up-regulated the expressions of TNF-R1, Fas, and Caspase8. Additionally, the expression changes of the intrinsic pathway-related proteins (Bak, Caspase9, Caspase3, and Bcl-xl) in JMFs-treated HepG2 cells were also detected. The results revealed that JMFs markedly promoted the expressions of Bak, Caspase9, and Caspase3, and down-regulated the expression of the anti-apoptotic protein (Bcl-xl) in HepG2 cells (Figure 10D). The original Western blot images were shown in Supplementary Materials Figure S2. Steps taken to present the results in Figure 10D were completed following the explanations provided in Figure S3 and Video S1.

**Figure 11:**
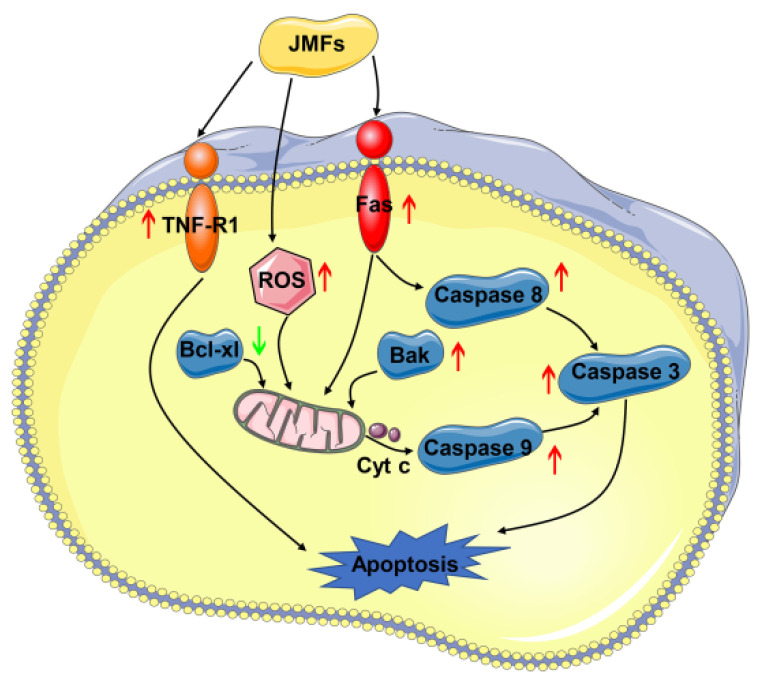
Proposed mechanism of apoptosis of HepG2 cells induced by JMFs.

In view of the key role of apoptosis in tumor treatment, the complex signal network that regulates apoptosis is gradually understood. It is noteworthy that the members of the Caspase family play a central role in cell apoptosis. Activating the initiator caspases (such as caspase-8 and -9) can lead to further activation of downstream effector caspases, such as caspase-3, -6, and -7 [44,45]. The activation of caspases is regulated by the extrinsic and intrinsic pathways. In the extrinsic pathway, TNF-R1 and Fas are the two key death domain-containing receptors binding to tumor necrosis factor (TNF) and Fas ligand (FasL), respectively, and their expression levels were found to be significantly up-regulated in the present study. Studies reported by others have shown that the up-regulated expressions of TNF-R1 and Fas can further activate downstream Caspases and induce the apoptosis of cells [46,47]. Herein, as the executor of apoptosis, the expression of Caspase8 was obviously up-regulated in JMFs-exposed cells. Accumulating evidence has shown that the up-regulated expression of Caspase8 is conducive to triggering the activation of Caspase3, further resulting in apoptosis. This effect of flavonoids from natural products has been described. Seon et al. found that the activities of flavonoids in Licorice in inducing the apoptosis of DU145 cells were achieved by up-regulating the expressions of FasL and caspase8 proteins [48]. Anti-apoptotic factor Bcl-xl plays an active role in the maintenance of mitochondrial integrity, while Bak plays the opposite role [49]. After Bak is released in cells subjected to internal stimulation, the mitochondria are destroyed to facilitate the release of cytochrome c, which further contributes to the activation of Caspase9 [50]. It has been reported that activated Caspase 9 is capable of further activating downstream Caspase 3, which interacts with Caspase8 and Caspase9 through the extrinsic and intrinsic pathways and induces apoptosis [51]. All of the above results suggest that the apoptosis of HepG2 cells induced by JMFs exposure may be achieved by affecting the expression regulation of related proteins in the extrinsic and intrinsic pathways (Figure 11). However, in addition to the two main pathways mentioned above, there may also be other pathways involved in JMFs-induced apoptosis, such as endoplasmic reticulum stress-induced apoptosis, which needs further research to be confirmed.

## 3. Materials and Methods

### 3.1. Materials and Chemicals

Waste branches of *J. mandshurica* were collected in July at Panshi, Jilin Province, China, dried at 45 °C, and ground to a fine powder (60 mesh) for future use. Macroporous resins (NKA-9, HPD-100, AB-8, DM130, and D101) were supplied by Beijing Rui Da Heng Hui Science & Technology Development Co. Ltd. (Beijing, China) and pretreated before use according to the method in the literature [20]. The physicochemical parameters of each resin are listed in Table S2 (Supplementary Materials).

Rutin standard (purity ≥ 98%) was supplied by Shanghai Yuanye Biotechnology Co., Ltd. (Shanghai, China). Chromatographic-grade and other analytical-grade chemicals were purchased from Sigma-Aldrich Co., Ltd. (St. Louis, MO, USA) and Sinopharm Chemical Reagent Co., Ltd. (Shanghai, China), respectively.

### 3.2. Preparation of the Crude Flavonoid Extracts from J. mandshurica

Ultrasonic-assisted extraction was introduced to prepare raw flavonoid extracts from the waste branches of *J. mandshurica* [52]. The supernatant was harvested as the raw extracts of flavonoids by centrifugation (8000× *g*, 10 min) and used to determine the amount of flavonoids using the method in the literature [53]. The standard curve was drawn with rutin as the standard substance, and the content of total flavonoids was calculated, which was expressed as the micrograms of rutin equivalents per gram of raw materials (dry weight).

### 3.3. Static Adsorption and Desorption Tests

Pretreated macroporous resin (6 g) and 80 mL of crude flavonoid extracts were mixed in a conical flask and then shaken for 24 h at 150 r/min and 25 °C. After filtration, the supernatant was introduced to determine the contents of flavonoids. Equation (1) was used to calculate the capacity of the resin to adsorb flavonoids.

Resin adsorbed with flavonoids was collected by filtration, and washed with distilled water, followed by desorption with ethanol solution at 150 r/min and 25 °C for 24 h. After filtration, the contents of JMFs presented in the eluent were measured and further used to calculate the desorption capacity and desorption ratio of the resin using Equation (2) and Equation (3), respectively [20].

(1) $$Q_{e}=\frac{\left(C_{0}-C_{e}\right)V_{Q}}{W}$$

(2) $$Q_{d}=\frac{C_{d}V_{d}}{W}$$

(3) $$D=\frac{Q_{d}}{Q_{e}}\times 100\%$$

The adsorption capacity (mg/g) is expressed as *Q_e_*. *C*_0_ and *C_e_* in the above formula represent the initial concentrations of the flavonoids and their equilibrium concentrations, respectively (mg/mL). *V_Q_* expresses the volume of the raw flavonoid solution (mL), and W is the weight of the resin used (g). *Q_d_* stands for the desorption capacity (mg/g). *C_d_* is the concentration of the flavonoids in the desorption solution (mg/mL). *V_d_* and *D* are the volume of the desorption solution (mL) and the desorption ratio of the resin, respectively.

### 3.4. Adsorption Kinetics

Crude flavonoid extracts (80 mL) were fully mixed with the pretreated resin (6 g) and adsorbed at 25 °C and 150 r/min for 10 h. During the whole adsorption process, the amount of flavonoids in the equilibrium solution at a fixed time (0.5, 1.0, 1.5, 2.0, 2.5, 3.0, 4.0, 5.0, 6.0, 7.0, 8.0, 10.0 h) was detected. Then, to explain the possible adsorption mechanism of the resin selected in this study, different kinetic models were employed to fit the adsorption process, including an intra-particle diffusion model, a pseudo-first-order model, and a pseudo-second-order model [29]. The three kinetic models mentioned above are represented by Equations (4)–(6), respectively.

(4) $$Q_{t}=k_{i}t^{1/2}+C$$

(5) $$\ln(Q_{e}-Q_{t})=-k_{1}t+\ln Q_{e}$$

(6) $$\frac{t}{Q_{t}}=\frac{1}{k_{2}Q_{e}^{2}}+\frac{t}{Q_{e}}$$

*Q_t_* represents the adsorption capacity (mg/g) of the resin at *t* (h). *C* expresses the kinetic model constant of the intra-particle diffusion model (mg/g). *k*_i_, *k*_1_, and *k*_2_ represent the adsorption rate constants of the intra-particle diffusion model [mg/(g·h^1/2^)], the pseudo-first-order model (h^−1^), and the pseudo-second-order model [g/(mg·h)], respectively.

### 3.5. Adsorption Isotherms

The flavonoid solutions (40 mL) with various concentrations (0.4, 0.6, 0.8, 1.0, 1.2, 1.4, 1.7, and 2.0 mg∙mL^−1^) were added to the conical bottle with the selected macroporous resin (3 g). Then, adsorption was carried out at different temperatures (25 °C, 35 °C, 45 °C) and a constant oscillation speed (150 r/min). After 24 h, the amounts of flavonoids in the equilibrium solutions were detected. Then, to describe the adsorption characteristics between the adsorbents and adsorbates, the adsorption results were fitted by the Langmuir model (7), Freundlich model (8), and Temkin isotherm model (9), respectively [29].

(7) $$\frac{C_{e}}{Q_{e}}=\frac{1}{Q_{m}K_{L}}+\frac{C_{e}}{Q_{m}}$$

(8) $$\ln Q_{e}=\frac{1}{n}\ln C_{e}+\ln K_{F}$$

(9) $$Q_{e}=B_{T}\ln K_{T}+B_{T}\ln C_{e}$$

where *Q_m_* is the saturated adsorption capacity (mg/g). *K_L_* (mL·mg^−1^), *K_F_* [(mg/g)(mL/mg)^1/n^] and *K_T_* (mL·mg^−1^) are the constants of the Langmuir models, Freundlich models, and Temkin models, respectively. 1/n is the adsorption strength of the adsorbent. *B_T_* represents the constant related to the heat of adsorption (J/mol).

### 3.6. Dynamic Adsorption and Desorption Tests

Considering the efficiency of purification, suitable adsorption and desorption conditions were selected. Shortly, after a glass column (2.0 cm × 35 cm, bed volume BV = 25 mL) filled with wet macroporous resin (AB-8) was equilibrated, different volumes of crude extracts were added and passed through the glass column at three flow rates (1.2, 2.4 and 3.6 BV/h). The flavonoid contents in the effluent were determined regularly. After the leakage curve was drawn, the loading volumes and loading flow rates of the samples were determined. Before the dynamic desorption process, a deionized water washing of the loaded resin was carried out to remove unabsorbed flavonoids and other compounds from the chromatographic column. Then, flavonoids were eluted from the loaded resin with an ethanol solution. Briefly, 20–100% ethanol solution was forced to pass through the glass column at different flow rates (1.2, 2.4, 3.6, 4.8 BV/h), and then the desorption solution was collected for the determination of flavonoids. Finally, the ethanol concentration, elution flow rate, and ethanol volume were determined. The purified total flavonoids (JMFs) were freeze-dried (Genesis2000SQ, VirTis, Gardener, NY, USA), and their purity was determined.

### 3.7. Fourier Transform Infrared Spectroscopy Analyses of JMFs

The infrared spectrum of JMFs in the region of 4000–400 cm^−1^ was detected by a Fourier transform infrared (FTIR) spectrometer (Thermo Nicolet IS 50, Madison, WI, USA) at a resolution of 4 cm^−1^, and the functional group of JMFs was obtained.

### 3.8. High-Performance Liquid Chromatography–Mass Spectrometry (HPLC-MS/MS)

The purified flavonoids were identified by HPLC-MS/MS according to the methods in previous studies [54]. The freeze-dried JMFs powder (100 mg) was fully dissolved in a methanol solution (800 μL), filtrated using a 0.22 μm diameter filter, and used for HPLC detection.

An Exion LC series HPLC (SCIEX, Framingham, MA, USA) equipped with a diode-array detector (DAD) system and an Xselect HSS T3 column (2.5 × 150 mm). Formic acid (0.1%) in water (*v*/*v*) was used as mobile phase A, while formic acid was added to the acetonitrile solution at a proportion of 1% (*v*/*v*), which was used as mobile B. A gradient elution program was adopted as follows: 0–15 min, 2% B; 15–17.1 min, 100% B; 17.1–20 min, 2% B. The oven temperature, flow rate, and injection volume of the samples were maintained at 50 °C, 0.4 mL/min, and 20 μL, respectively.

Triple quadrupole tandem mass spectrometry was carried out on a QTRAP 6500+ mass spectrometer (SCIEX, USA) with an electrospray ionization (ESI) interface. The ESI source was in the positive and negative ion mode. The detection settings were as follows: source temperature of 550 °C, ion spray voltages of +5.5 kV and −4.5 kV, ion source gas pressure of 0.41 MPa, curtain gas pressure of 0.24 MPa, auxiliary air pressure of 0.41 MPa, the collision energy of 35 NCE. The flavonoids in the samples were qualitatively and relatively quantitatively analyzed by multiple reaction monitoring.

### 3.9. Antitumor Efficiency of JMFs

#### 3.9.1. Culture Conditions of Cells Used in the Present Study

Human hepatoma HepG2 cell line and human normal hepatic cells (HL-7702 cells), provided by Jilin University (Changchun, China), were routinely cultured in Dulbecco’s Modified Eagle’s Medium (DMEM) supplemented with penicillin/streptomycin (1%) and fetal bovine serum (FBS, 10%) at 37 °C in an incubator with 5% CO_2_ in the air.

#### 3.9.2. Cell Viability Detection by CCK8 Kit

A cell counting kit 8 (Solarbio, Beijing, China) was used to determine the viability of the HepG2 cells [34]. In short, the cells were seeded onto 96-well plates at a density of 5 × 10^3^ cells/well and allowed to grow at 37 °C. Then, 12 h later, the cells were administered with a complete medium containing 0.1% DMSO (as a negative control) and different concentrations of JMFs (10, 20, 40, 60, 80, 100, 120, and 140 μg/mL) and further incubated for 1 d and 2 d, respectively. Subsequently, after the removal of the original medium, 100 μL of fresh serum-free DMEM medium was added and incubated with CCK8 reagent (10 μL) for 75 min at 37 °C. The absorbance was detected at 450 nm with the help of a microplate reader (Tecan, Spark, Switzerland). Four replicates were performed for each treatment. The values of an inhibition concentration of 50% (IC_50_) were determined by GraphPad Prism 8.0.2 software (GraphPad software Inc., San Diego, CA, USA).

#### 3.9.3. Observation of Cell Morphology

After 48 h of the JMFs (0, 40, 80, and 120 μg/mL) treatment, the cells were stained with or without acridine orange, and their morphology was observed with an inverted fluorescence microscope (Olympus IX51, Tokyo, Japan).

#### 3.9.4. Colony-Forming Assay

The effect of JMFs on the colony forming of HepG2 cells was also investigated as described previously [55]. In brief, cell suspensions (500 cells/well) were added to plates with 6 wells (Corning Inc., Corning, NY, USA) in triplicate and routinely cultured overnight. Then, the cells were exposed to JMFs (0, 20, 40, and 60 μg/mL) for 48 h. Subsequently, after washing with phosphate-buffered saline (PBS) 3 times, the cells were incubated in the medium without reagent for another 10 days. The culture was terminated once the number of cells in the colony was greater than or equal to 50. Then, the colonies were fixed with paraformaldehyde (4%, 15 min) and stained with crystal violet (0.1%, 15 min). After the dye removal, water rinsing, and drying, the number of colonies was recorded.

#### 3.9.5. Migration and Invasion Assay

Cells were inoculated on 6-well plates at a density of 3 × 10^5^ cells per well in a humidified air incubator at 37 °C for 48 h. Following the discarding of the culture medium, the plates were washed as described above. Wounds were formed with an aseptic 200 μL microtip. Afterward, JMFs (0, 40, 80, and 120 μg/mL) were added. After 48 h of treatment, images of each well were observed with an inverted microscope (Olympus IX51, Tokyo, Japan) and analyzed by ImageJ 1.52a software (National Institutes of Health, Bethesda, MD, USA). Then, the migration percentage of the cells was calculated.

A transwell chamber test was employed to detect the invasive ability of cells, following the method reported by Shi et al. [56]. Briefly, the upper chamber was covered with 100 μL of diluted Matrigel (BD Biosciences, San Jose, CA, USA) and put into an incubator at 37 °C until solidification. Serum-free DMEM containing different concentrations of JMFs was employed to resuspend the HepG2 cells until the cell density reached 2 × 10^5^ cells/mL. Then, the above suspension (200 μL) was taken out and placed in the upper chamber. At the same time, the lower chamber was added with DMEM containing 10% FBS (700 μL). Following incubation for 48 h under the conditions mentioned above, the upper chamber was treated with cotton swabs to remove the cells. After a series of treatments (fixation with 4% formaldehyde for 15 min, washing with PBS twice, staining with crystal violet for 15 min, washing with distilled water), the cells located on the lower surface of the chambers were observed using a microscope (Leica, Wetzlar, Germany).

#### 3.9.6. Cell Cycle Analysis

The distribution of the cell cycle was detected using a cell cycle assay kit (Solarbio, Beijing, China). Briefly, HepG2 cells were cultured and incubated with selected samples according to the method mentioned above. After the cells treated for 48 h were collected, ice-cold PBS washing and 75% ethanol fixation (24 h, 4 °C) were carried out in turn. Then, following the washing of the fixed cells (1 × 10^6^ cells/mL) with PBS again, 100 mL of RNase A was introduced and incubated with the cells for half an hour at 37 °C. Subsequently, the staining treatment was carried out with propidium iodide (PI) solution (50 μL). Half an hour later, a flow cytometer (LSR fortessa, BD Biosciences, Franklin Lakes, NJ, USA) was used for the detection of the cell cycle distribution.

#### 3.9.7. Determination of Reactive Oxygen Species (ROS)

To investigate the changes in the intracellular ROS generation of HepG2 cells, an ROS assay kit (Solarbio, Beijing, China) was used. In short, HepG2 cells and different substances were placed in plates with 6 wells and incubated for 48 h, stained with 2 mL of DCFH-DA at 37 °C in the dark (0.5 h), and washed with PBS. A fluorescence microscope (Olympus IX51, Tokyo, Japan) was introduced to observe the cells immediately, followed by an analysis of the fluorescence intensity detected by flow cytometry (LSR fortessa, BD Biosciences, Franklin Lakes, NJ, USA).

#### 3.9.8. Apoptosis Was Detected by Hoechst 33342 Staining

Hoechst 33342 staining was carried out as in a previous study with a minor revision [13]. Briefly, JMFs-treated HepG2 cells were washed with PBS twice, followed by staining with Hoechst 33342 solution (10 μg/mL) at 37 °C for 20 min. Finally, the cells were observed under a fluorescence microscope.

#### 3.9.9. Annexin V-FITC/PI Staining

The apoptosis of HepG2 cells induced by JMFs was further detected using an Annexin V-FlTC apoptosis detection kit (Solarbio, Beijing, China). As indicated in the instructions, HepG2 cells inoculated at a density of 2 × 10^5^ cells/well for 12 h were further incubated with JMFs (0, 40, 80, 120 μg/mL) for 48 h. Afterward, the collected cells were resuspended in a binding buffer to a concentration of 1 × 10^6^ cells/mL. Then, cell staining with Annexin V-FlTC/PI was carried out in the dark (15 min, room temperature). The rate of apoptotic cells was detected using a flow cytometry system (LSR fortessa, BD Biosciences, Franklin Lakes, NJ, USA).

### 3.10. Western Blot

The total proteins were isolated from the cell lysates using RIPA buffer (Solarbio, Beijing, China) referring to the instructions. The total protein concentration was determined using the bicinchoninic acid assay (BCA assay). After separation with sodium dodecyl sulfate polyacrylamide gel electrophoresis (SDS-PAGE), the proteins were transferred onto polyvinylidene fluoride (PVDF) membranes (Millipore), which were further blocked with 5% nonfat milk for 2 h and subsequently incubated with primary antibodies against Caspase 8, Caspase 9, Caspase 3, TNF-R1, Fas, Bcl-xl, Bak, and β-actin (Bioss, 1:1000, Beijing, China) at 4 °C for 12 h. Subsequently, an anti-rabbit horseradish peroxidase-conjugated antibody (Bioss, 1:1000, Beijing, China) was introduced to continue the incubation for 1.5 h. Then, a blocking buffer was used for the treatment of the membrane (30 min, 3 times). Finally, Quantity One software (Bio Rad Laboratories, Richmond, CA, USA) was used for taking photos and determining the statistics. In order to serve as an internal control, β-Actin was introduced.

### 3.11. Statistical Analysis

The above experiments were repeated more than 3 times, and their results were expressed as the mean ± standard deviation (S.D.). For determining the statistical significance, one-way analysis of variance (ANOVA), followed by post hoc multiple comparisons (Turkey’s test) was employed using GraphPad 8.0.2 Software (GraphPad, San Diego, CA, USA). Statistically significant differences were identified when the *p*-value was not greater than 0.05.

## 4. Conclusions

Overall, in the present study, AB-8 resin was chosen for the purification of JMFs from *J. mandshurica*. The equilibrium adsorption process of the AB-8 resin toward JMFs was well suited to the pseudo-second-order kinetic model and the Langmuir isotherm model. Furthermore, suitable purification parameters were obtained by dynamic adsorption/desorption experiments. In addition, flavonoids extracted from *J. mandshurica* can not only inhibit the proliferation of HepG2 cells but also affect the migration and invasion of HepG2 cells. The inhibitory effects of JMFs on HepG2 cells may be achieved by inducing the production of ROS, apoptosis, and cell cycle arrest. Our primary investigation into the anti-hepatoma activities of flavonoids obtained from *J. mandshurica* will benefit the isolation of active principles responsible for antitumor effects. However, more in vivo studies are needed to understand the antitumor mechanism of JMFs and confirm their broad-spectrum anticancer activity.

## Figures and Tables

**Table 1:** Adsorption kinetics models and related parameters of AB-8 resin towards flavonoids extracted from *J. mandshurica*.

Kinetics Models	Equations	Dynamic Parameters
Intra-particle diffusion	$Q_{t}=0.4796t^{1/2}+5.729$	*k_i_* = 0.4796 mg/(g·h^1/2^) *C* = 5.729 mg/g *R*^2^ = 0.6140
Pseudo-first-order	$\ln(Q_{e}-Q_{t})=-0.4989t-0.4184$	*k*_1_ = 0.4989 h^−1^ *Q_e_* = 0.07 mg/g *R*^2^ = 0.6239
Peudo-second-order	$\frac{t}{Q_{t}}=0.1421t+0.01633$	*k*_2_ = 1.2365 g/(mg·h) *Q_e_* = 7.04 mg/g *R*^2^ = 0.9999

**Table 2:** The adsorption isotherms and parameters of AB-8 resin towards flavonoids extracted from *J. mandshurica* at different temperatures.

Models	*T* (°C)	Equations	Parameters		
			*K_L_* (mL/mg)	*Q_m_* (mg/g)	*R* ^2^
Langmuir	25	$\frac{C_{e}}{Q_{e}}=0.0793C_{e}+0.0054$	14.69	12.61	0.9973
	35	$\frac{C_{e}}{Q_{e}}=0.0808C_{e}+0.0111$	7.28	12.38	0.9965
	45	$\frac{C_{e}}{Q_{e}}=0.0851C_{e}+0.0162$	5.25	11.75	0.9960
			*K_F_* [(mg/g)(mL/mg)^1/n^]	1/n	*R* ^2^
Freundlich	25	$\ln Q_{e}=0.2916\ln C_{e}+2.591$	13.34	0.2916	0.8417
	35	$\ln Q_{e}=0.3525\ln C_{e}+2.471$	11.83	0.3525	0.8962
	45	$\ln Q_{e}=0.3819\ln C_{e}+2.351$	10.50	0.3819	0.9014
			*K_T_* (mL/mg)	*B_T_* (J/mol)	*R* ^2^
Temkin	25	$Q_{e}=2.382\ln C_{e}+12.64$	201.64	2.382	0.8911
	35	$Q_{e}=2.622\ln C_{e}+11.31$	74.70	2.622	0.9414
	45	$Q_{e}=2.598\ln C_{e}+10.13$	49.36	2.598	0.9487

**Table 3:** Structure backbone of flavonoids isolated from *J. mandshurica*.

Group of Flavonoids	Structure Backbone	Examples	
chalcones	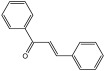	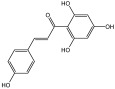	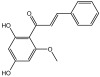
		naringenin chalcone	cardamonin
dihydrochalcones	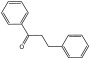	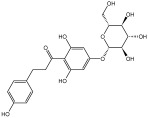	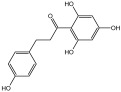
		trilobatin	phloretin
flavanones	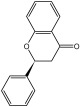	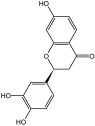	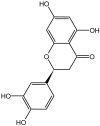
		butin	eriodictyol
flavones	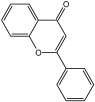	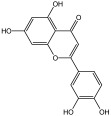	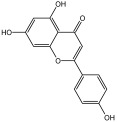
		luteolin	apigenin
flavonols	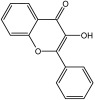	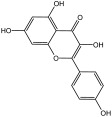	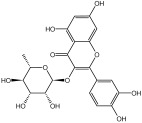
		kaempferol	quercitrin
anthocyanins	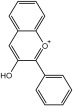	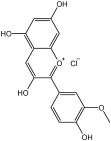	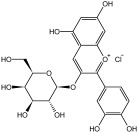
		peonidin chloride	idaein chloride
isoflavonoids	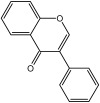	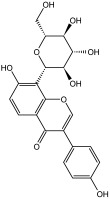	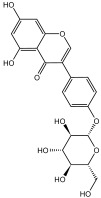
		puerarin	sophoricoside

## Data Availability

Data presented in the present study are available on request from the corresponding author.

## Supplementary Materials

The following supporting information can be downloaded at: https://www.mdpi.com/article/10.3390/molecules29091976/s1, Table S1: Flavonoids in JMFs were identified by HPLC-MS/MS; Table S2: Physicochemical properties of the macroporous resins used in this study; Figure S1: The effects of JMFs on the proliferation of HL-7702 cells; Figure S2. The original Western blot images for panels in Figure 10. The areas within the red boxes are cropped and presented in Figure 10C,D; Figure S3. Steps taken to obtain Figure 10D; Video S1—Processing of raw WB images.
