# Supplementary material for: Enrichment and Evaluation of Antitumor Properties of Total Flavonoids from Juglans mandshurica Maxim
Source: Molecules. 2024 Apr 25;29(9):1976. doi: 10.3390/molecules29091976 (PMC11085465; doi:10.3390/molecules29091976)
Supplement: Supplementary file 1 [file molecules-29-01976-s001.zip › molecules-2171640-supplementary.pdf]

Supplementary materials

Table S1. Flavonoids in JMFs were identified by HPLC-MS/MS

No.	Compound name	Predicted formula	Retention time (min)	Relative molecular weight	Peak area
**chalcones and dihydrochalcones (6)**					
1	naringenin chalcone	$C_{15}H_{12}O_5$	8.52	272.253	216500000
2	butein	$C_{15}H_{12}O_5$	0.95	272.069	9251
3	trilobatin	$C_{21}H_{24}O_{10}$	7.52	436.409	5249000
4	isoliquiritigenin	$C_{15}H_{12}O_4$	9.23	256.25	4001
5	cardamonin	$C_{16}H_{14}O_4$	11.62	270.28	15330
6	phloretin	$C_{15}H_{14}O_5$	8.60	274.27	412200
**flavanones (21)**					
7	eriodictyol	$C_{15}H_{12}O_6$	8.00	288.252	13910000
8	taxifolin O-glucoside	$C_{21}H_{22}O_{12}$	5.31	466.392	10670000
9	neohesperidin	$C_{28}H_{34}O_{15}$	7.20	610.56	241900
10	naringin	$C_{27}H_{32}O_{14}$	6.98	580.53	808600
11	narirutin	$C_{27}H_{32}O_{14}$	6.84	580.535	1084000
12	butin	$C_{15}H_{12}O_5$	8.61	272.069	172000000
13	hesperidin	$C_{28}H_{34}O_{15}$	7.09	610.56	195300
14	hesperetin	$C_{16}H_{14}O_6$	8.97	302.27	103600
15	liquiritigenin	$C_{15}H_{12}O_4$	8.90	256.25	2964
16	purpurin	$C_{14}H_8O_5$	10.55	256.21	874800
17	liquiritin	$C_{21}H_{22}O_9$	6.98	418.39	12690
18	naringenin	$C_{15}H_{12}O_5$	8.68	272.25	153200000
19	pinocembrin	$C_{15}H_{12}O_4$	10.37	256.25	14150
20	neoeriocitrin	$C_{27}H_{32}O_{15}$	6.38	596.542	2371000
21	poncirin	$C_{28}H_{34}O_{14}$	8.05	594.561	487100
22	bavachin	$C_{20}H_{20}O_4$	10.72	324.37	4322
23	phellodensin F	$C_{26}H_{30}O_{10}$	7.01	502.2	411100
24	hesperetin 5-O-glucoside	$C_{22}H_{24}O_{11}$	6.59	464.419	63900000
25	prunin	$C_{21}H_{22}O_{10}$	7.02	434.121	39270000
26	naringerin	$C_{15}H_{12}O_5$	8.64	272.253	209200000
27	fustin	$C_{15}H_{12}O_6$	5.89	288.252	62390000
**flavones and flavonols (43)**					
28	isoschaftoside	$C_{26}H_{28}O_{14}$	6.03	564.499	11700
29	myricitrin	$C_{21}H_{20}O_{12}$	6.55	464.38	6488000
30	azaleatin	$C_{16}H_{12}O_7$	7.30	316.265	1584000

No.	Compound name	Predicted formula	Retention time (min)	Relative molecular weight	Peak area
31	apigenin	$C_{15}H_{10}O_5$	8.70	270.24	587300
32	kaempferol	$C_{15}H_{10}O_6$	8.81	286.24	1406000
33	luteolin	$C_{15}H_{10}O_6$	8.06	286.24	240200
34	baicalein	$C_{15}H_{10}O_5$	9.18	270.24	8831
35	myricetin	$C_{15}H_{10}O_8$	7.34	318.24	13230000
36	quercitrin	$C_{21}H_{20}O_{11}$	7.24	448.38	5939000
37	quercetin	$C_{15}H_{10}O_7$	8.96	302.236	870600
38	quercetin-3´-O-glucoside	$C_{21}H_{20}O_{12}$	6.53	464.382	1684000
39	3´-hydroxyflavone	$C_{15}H_{10}O_3$	10.05	238.24	44830
40	hyperoside	$C_{21}H_{20}O_{12}$	6.55	464.38	4995000
41	isovitexin	$C_{21}H_{20}O_{10}$	6.53	432.378	10310
42	tiliroside	$C_{30}H_{26}O_{13}$	7.88	594.52	20790
43	isorhamnetin	$C_{16}H_{12}O_7$	8.97	316.262	37280
44	rutin	$C_{27}H_{30}O_{16}$	6.45	610.518	40510
45	vitexin	$C_{21}H_{20}O_{10}$	6.56	432.38	7166
46	wogonin	$C_{16}H_{12}O_5$	10.29	284.26	4223
47	diosmetin	$C_{16}H_{12}O_6$	8.91	300.26	180500
48	scutellarein	$C_{15}H_{10}O_6$	7.64	286.236	11030
49	astragalin	$C_{21}H_{20}O_{11}$	7.24	448.38	5270000
50	syringetin	$C_{17}H_{14}O_8$	7.87	346.069	70860
51	kaempferol 7-O-rhamnoside	$C_{21}H_{20}O_{10}$	7.36	432.106	1499000
52	laricitrin	$C_{16}H_{12}O_8$	2.60	332.262	2480000
53	isoquercitrin	$C_{21}H_{20}O_{12}$	6.60	464.38	1143000
54	vaccarin	$C_{32}H_{38}O_{19}$	5.90	726.633	6674
55	epimedin A	$C_{39}H_{50}O_{20}$	7.80	838.802	9121
56	mosloflavone	$C_{17}H_{14}O_5$	11.74	298.295	275700
57	camelliaside A	$C_{33}H_{40}O_{20}$	6.31	756.668	33020
58	myricetin 3-O-galactoside	$C_{21}H_{20}O_{13}$	7.19	480.376	596500
59	neodiosmin	$C_{28}H_{32}O_{15}$	7.12	608.545	411800
60	toringin	$C_{21}H_{20}O_9$	6.23	416.378	300200
61	moslosooflavone	$C_{17}H_{14}O_5$	11.74	298.295	204200
62	ladanein	$C_{17}H_{14}O_6$	8.06	314.295	17180
63	isosinensetin	$C_{20}H_{20}O_7$	9.37	372.375	27940
64	icariin	$C_{33}H_{40}O_{15}$	8.14	676.66	2645
65	nobiletin	$C_{21}H_{22}O_8$	10.52	402.39	271700
66	sinensetin	$C_{20}H_{20}O_7$	9.55	372.37	26320
67	tangeretin	$C_{20}H_{20}O_7$	11.41	372.37	102100
68	isohyperoside	$C_{21}H_{20}O_{12}$	5.91	464.095	15160000

No.	Compound name	Predicted formula	Retention time (min)	Relative molecular weight	Peak area
69	pectolinarigenin	$C_{17}H_{14}O_6$	10.78	314.295	15140
70	quercetin-O-glucoside	$C_{21}H_{20}O_{12}$	6.54	464.376	48130000
**flavonoids (59)**					
71	apigenin 5-O-glucoside	$C_{21}H_{20}O_{10}$	6.68	432.38	193300
72	luteolin-7-O-beta-D-glucuronide	$C_{21}H_{18}O_{12}$	6.53	462.366	327700
73	ophiopogonanone C	$C_{19}H_{16}O_7$	5.38	356.332	1585000
74	apigenin O-hexosyl-O-pentoside	$C_{26}H_{28}O_{14}$	6.83	564.1	210400
75	methyl-Hesperidin	$C_{29}H_{38}O_{16}$	7.57	642.59	53470
76	hydroxygenkwanin	$C_{16}H_{12}O_6$	9.60	300.263	22750
77	engeletin	$C_{21}H_{22}O_{10}$	7.15	434.393	88690
78	apigenin 4-O-rhamnoside	$C_{21}H_{20}O_9$	7.97	416	54720
79	amentoflavone	$C_{30}H_{18}O_{10}$	9.16	538.46	85320
80	apigenin7-O-beta-D-glucuronide	$C_{21}H_{18}O_{11}$	7.08	446.3670044	32840
81	kuwanon A	$C_{25}H_{24}O_6$	0.70	420.454	166100
82	wogonoside	$C_{22}H_{20}O_{11}$	8.26	460.388	1706000
83	spiraeoside	$C_{21}H_{20}O_{12}$	6.59	464.096	8312000
84	saponarin	$C_{27}H_{30}O_{15}$	6.27	594.518	26010
85	isotrifoliin	$C_{21}H_{20}O_{12}$	6.59	464.096	8881000
86	kaempferin	$C_{21}H_{20}O_{10}$	7.35	432.106	1496000
87	Di-O-methylquercetin	$C_{17}H_{14}O_7$	9.19	330.1	25780000
88	kaempferol 3-A-L-arabinopyranoside	$C_{20}H_{18}O_{10}$	7.08	418.351	4677
89	pinobanksin	$C_{15}H_{12}O_5$	7.06	272.069	73420000
90	afzelechin	$C_{15}H_{14}O_5$	0.68	274.084	184900
91	anhydroicaritin	$C_{21}H_{20}O_6$	12.67	368.38	34120
92	epicatechin Gallate	$C_{22}H_{18}O_{10}$	6.54	442.379	14500000
93	phlorizin	$C_{21}H_{24}O_{10}$	7.24	436.41	6545000
94	astilbin	$C_{21}H_{22}O_{11}$	6.75	450.39	168500
95	heptamethoxyflavone	$C_{22}H_{24}O_9$	7.08	432.42	73890
96	vincetoxicoside B	$C_{21}H_{20}O_{11}$	6.97	448.383	2285000
97	(-)-gallocatechin	$C_{15}H_{14}O_7$	4.51	306.267	672800
98	taxifolin	$C_{15}H_{12}O_7$	6.89	304.25	82890000
99	silymarin	$C_{25}H_{22}O_{10}$	8.51	482.44	59560
100	isorhoifolin	$C_{27}H_{30}O_{14}$	6.84	578.52	7592
101	scutellarin	$C_{21}H_{18}O_{12}$	6.59	462.36	37440
102	rutin hydrate	$C_{27}H_{30}O_{16}\cdot H_2O$	6.36	610.52	30210

No.	Compound name	Predicted formula	Retention time (min)	Relative molecular weight	Peak area
103	dihydromyricetin	$C_{15}H_{12}O_8$	6.97	320.25	5322000
104	morin	$C_{15}H_{10}O_7$	8.05	302.043	33970000
105	dihydrokaempferol	$C_{15}H_{12}O_6$	5.75	288.063	17930000
106	7-O-methyleriodictyol	$C_{16}H_{14}O_6$	8.86	302.079	56400
107	7-hydroxyflavone	$C_{15}H_{10}O_3$	9.17	238.24	2198
108	isomucronulatol-7-O-glucoside	$C_{23}H_{28}O_{10}$	6.53	464.469	5360000
109	chrysoeriol 5-O-hexoside	$C_{22}H_{22}O_{11}$	7.18	462.404	229400
110	kaempferol-3-gentiobioside	$C_{27}H_{30}O_{16}$	6.28	610.525	38800
111	narcissoside	$C_{28}H_{32}O_{16}$	6.82	624.544	118300
112	apigenin C-glucoside	$C_{21}H_{20}O_{10}$	6.46	432.113	70130
113	irisolidone 7-O-beta-d-glucoside	$C_{23}H_{24}O_{11}$	0.97	476.43	8800
114	apigenin-6,8-di-C-glycoside	$C_{27}H_{30}O_{15}$	5.73	594.518	125600
115	5,7-dihydroxy-3′,4′,5′-trimethoxyflavone	$C_{18}H_{16}O_7$	4.99	344.09	306000
116	chalcone	$C_{15}H_{12}O$	6.47	208.255	510100
117	5-methoxyflavone	$C_{16}H_{12}O_3$	10.47	252.26	260400
118	chrysoeriol 7-O-hexoside	$C_{22}H_{22}O_{11}$	7.20	448.4	307800
119	luteolin-4′-O-glucoside	$C_{21}H_{20}O_{11}$	6.80	448.101	150600000
120	O-methylnaringenin C-pentoside	$C_{21}H_{22}O_9$	0.72	418.1	144700
121	columbianetin	$C_{14}H_{14}O_4$	6.86	246.263	31530
122	homoplantaginin	$C_{22}H_{22}O_{11}$	7.19	462.403	337300
123	4′-O-glucosylvitexin	$C_{27}H_{30}O_{15}$	6.28	594.518	253000
124	eriodictyol-7-O-glucoside	$C_{21}H_{22}O_{11}$	5.77	450.393	12610000
125	troxerutin	$C_{33}H_{42}O_{19}$	6.57	742.68	16210
126	linarin	$C_{28}H_{32}O_{14}$	7.79	592.553	238900
127	kaempferol-3-O-rutinoside	$C_{27}H_{30}O_{15}$	6.51	594.526	222500
128	oroxin A	$C_{21}H_{20}O_{10}$	7.56	432.378	337200
129	rhamnetin	$C_{16}H_{12}O_7$	6.88	316.262	305300
	**anthocyanins (13)**				
130	procyanidin B2	$C_{30}H_{26}O_{12}$	5.16	578.529	265500
131	petunidin-3-O-glucoside chloride	$C_{22}H_{23}O_{12}Cl$	5.28	514.865	85160

No.	Compound name	Predicted formula	Retention time (min)	Relative molecular weight	Peak area
132	procyanidin B3	$C_{30}H_{26}O_{12}$	5.15	578.142	151500
133	rhein	$C_{15}H_8O_6$	10.11	284.225	631300
134	myrtillin chloride	$C_{21}H_{21}ClO_{12}$	8.52	500.84	36160
135	callistephin chloride	$C_{21}H_{21}ClO_{10}$	5.44	468.84	3218
136	idaein chloride	$C_{21}H_{21}ClO_{11}$	5.21	484.84	16360000
137	peonidin chloride	$C_{16}H_{13}ClO_6$	11.42	336.72	31910
138	cyanidin 3-O-glucoside	$C_{21}H_{21}ClO_{11}$	5.21	484.84	20280000
139	rhein 8-Glucoside	$C_{21}H_{18}O_{11}$	7.10	446.3670044	61750
140	delphinidin 3-O-rutinoside	$C_{27}H_{31}O_{16}$	6.35	611.5	20000
141	petunidin 3-O-glucoside	$C_{22}H_{23}O_{12}$	0.98	479	188900
142	cyanidin O-rutinoside	$C_{27}H_{31}O_{15}$	6.66	595.52	94820
**isoflavonoids (14)**					
143	prunetin	$C_{16}H_{12}O_5$	6.77	284.069	13210
144	orobol	$C_{15}H_{10}O_6$	11.19	286.048	63280
145	glabridin	$C_{20}H_{20}O_4$	11.47	324.37	1467
146	puerarin	$C_{21}H_{20}O_{10}$	5.75	432.38	12970
147	2´-hydroxydaidzein	$C_{15}H_{10}O_5$	7.24	270.053	109600
148	2´-hydroxygenistein	$C_{15}H_{10}O_6$	8.02	286.048	126200
149	medicarpin	$C_{16}H_{14}O_4$	7.26	270.285	44610
150	protobioside	$C_{45}H_{74}O_{18}$	7.89	903.071	71930
151	sissotrin	$C_{22}H_{22}O_{10}$	6.23	446.121	974500
152	deguelin	$C_{23}H_{22}O_6$	12.14	394.42	51040
153	6´´-O-xylosyl-glycitin	$C_{27}H_{30}O_{14}$	6.30	578.519	26220
154	ipriflavone	$C_{18}H_{16}O_3$	12.60	280.32	28510
155	sophoricoside	$C_{21}H_{20}O_{10}$	7.04	432.38	589400
156	ononin	$C_{22}H_{22}O_9$	1.04	430.405	14520

Table S2. Physicochemical properties of the macroporous resins used in this study

Resins	Particle size (mm)	Surface area (m^2/g)	Average pore diameter (nm)	Polarity
NKA-9	0.3-1.25	170-250	15.5-16.5	Polar
AB-8	0.3-1.25	450-530	13.0-14.0	Weak polar
DM130	0.3-1.25	500-550	9.0-10.0	Weak polar
HPD-100	0.3-1.25	480-520	25.0-28.0	Non-polar
D101	0.3-1.25	500-600	21.0-23.0	Non-polar

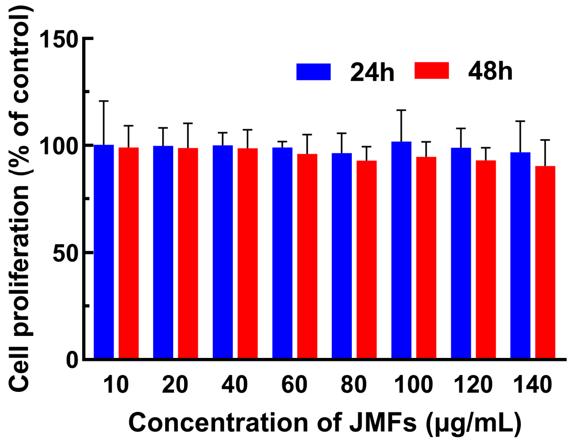

Figure S1. The effects of JMFs on the proliferation of HL-7702 cells.

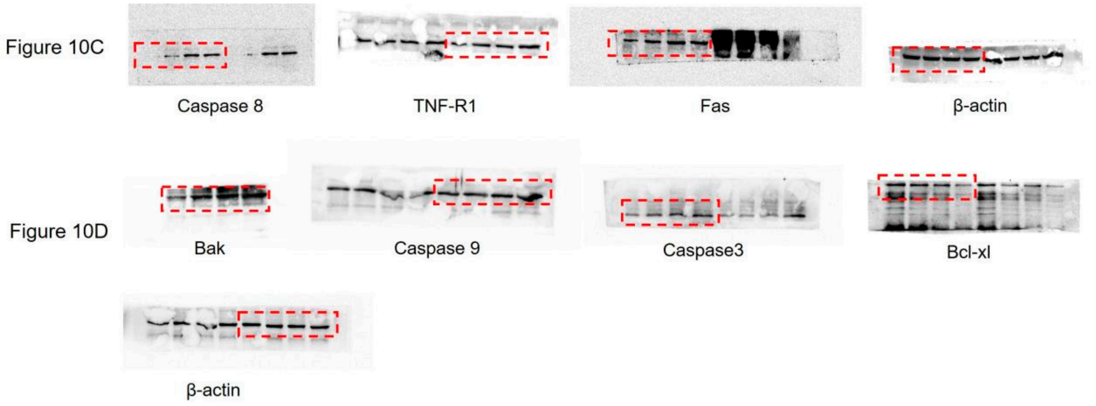

Figure S2. The original Western blot images for panels in Figure 10. The areas within the red boxes are cropped and presented in Figure 10C, and 10D.

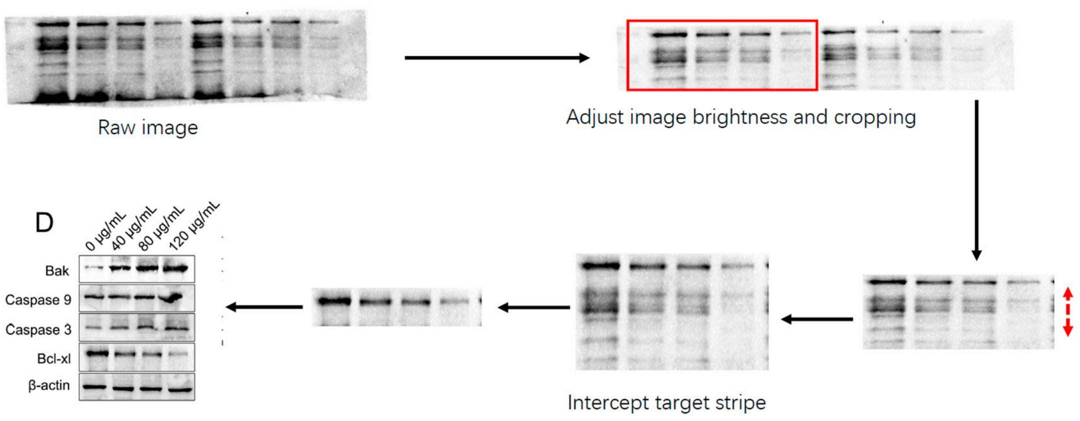

Figure S3. Steps taken to obtain Figure 10D.
